# Big data analysis of influenza vaccination and liver cancer risk in hypertensive patients: insights from a nationwide population-based cohort study

**DOI:** 10.1186/s12876-025-03665-w

**Published:** 2025-02-24

**Authors:** Chun-Chih Chiu, Wen-Rui Hao, Kuan-Jie Lin, Chun-Chao Chen, Tsung-Yeh Yang, Yu-Ann Fang, Tsung-Lin Yang, Yu-Hsin Lai, Ming-Yao Chen, Min-Huei Hsu, Cheng-Hsin Lin, Hsin Hsiu, Huan-Yuan Chen, Tzu-Hurng Cheng, Nai-Hsuan Chen, Ju-Chi Liu

**Affiliations:** 1 Division of Cardiology, Department of Internal Medicine, Shuang Ho Hospital, Taipei Medical University, New Taipei City, 23561 Taiwan; 2 Taipei Heart Institute, Taipei Medical University, Taipei, Taiwan; 3 Division of Cardiology, Department of Internal Medicine, School of Medicine, College of Medicine, Taipei Medical University, Taipei, Taiwan; 4 Graduate Institute of Medical Sciences, College of Medicine, Taipei Medical University, Taipei, 110 Taiwan; 5 Division of Cardiovascular Surgery, Department of Surgery, Shuang Ho Hospital, Taipei Medical University, New Taipei City, Taiwan; 6 Division of Cardiology, Department of Internal Medicine, Cardiovascular Research Center, Taipei Medical University Hospital, Taipei, 110 Taiwan; 7 Division of Gastroenterology and Hepatology, Department of Internal Medicine, School of Medicine, College of Medicine, Taipei Medical University, Taipei, Taiwan; 8 TMU Research Center for Digestive Medicine, Taipei Medical University, Taipei, 110 Taiwan; 9 Division of Gastroenterology and Hepatology, Department of Internal Medicine, Shuang Ho Hospital, New Taipei, Taiwan; 10 Graduate Institute of Data Science, College of Management, Taipei Medical University, Taipei, Taiwan; 11 Department of Neurosurgery, Shuang Ho Hospital, Taipei Medical University, New Taipei City, Taiwan; 12 Graduate Institute of Biomedical Engineering, National Taiwan University of Science and Technology, No.43, Section 4, Keelung Road, Taipei, 10607 Taiwan; 13 Institute of Biomedical Sciences, Academia Sinica, Taipei, 11578 Taiwan; 14 Department of Biochemistry, School of Medicine, College of Medicine, China Medical University, Taichung City, 404333 Taiwan; 15 Department of Physical medicine and rehabilitation, Wan Fang Hospital, Taipei Medical University, Taipei, Taiwan

**Keywords:** Hypertension, Influenza vaccination, Liver cancer

## Abstract

**Background:**

previous studies have indicated that influenza vaccination may be associated with reduced risks of certain types of cancer. However, the protective effect of influenza vaccination against primary liver cancer in individuals with hypertension remains unclear.

**Methods:**

In this cohort study, 37,022 patients over 55 years of age who received a diagnosis of hypertension at any time between January 1, 2001, and December 31, 2012, were enrolled from the National Health Insurance Research Database. The patients were divided into a vaccinated and an unvaccinated group. Categorical and continuous variables were analyzed using the chi-square test and t test, respectively, and the correlation between influenza vaccination and liver cancer in patients with hypertension was analyzed using time-varying COX model. Propensity score method was performed to reduce selection bias.

**Results:**

Compared with the unvaccinated group, the vaccinated group had a significantly lower incidence of liver cancer (hazard ratio = 0.56, 95% confidence interval = 0.46–0.64; *p* < .001). In addition, a protective effect was observed regardless of sex, age, or comorbidities. Besides, the association was dose-dependent which could be noted when patients were stratified based on the total number of vaccinations. The adjusted HRs for patients receiving 1, 2 to 3, and ≥ 4 vaccinations during the follow-up period were 0.60 (0.51–0.78), 0.48 (0.38–0.65), and 0.39(0.30–0.51), respectively.

**Conclusions:**

In summary, influenza vaccination is linked to a decreased risk of liver cancer in individuals with hypertension. However, unmeasurable confounders may have been present in the analysis.

## Background

Hypertension is a chronic disease that is serious and highly prevalent, especially given aging populations worldwide. More than 1 billion people worldwide have been estimated to have hypertension [1, 2]. In Taiwan, hypertension has a prevalence of 24.1% [3]. Elevated blood pressure is a risk factor for several conditions, such as cardiovascular disease, chronic kidney disease, and stroke [4]. Hypertension is also associated with an increased risk of cancer [5, 6].

Primary liver cancer is a common cancer in the world, especially in Taiwan [7]. According to the Health Promotion Administration, the age-standardized incidence rate of liver cancer in Taiwan was 26.14 per 100,000 individuals in 2020, which is significantly above than the global average (9.3 per 100,000 individuals) [8, 9]. Liver cancer is the second leading cause of cancer-related death in Taiwan, with a standardized mortality rate of 17.9 per 100,000 individuals [10]. According to previous studies, many risk factors have been associated with the incidence of liver cancer, such as infection with the hepatitis virus, exposure to aflatoxins, cigarette consumption, drinking alcohol, and diabetes [11–13]. Elevated blood pressure has also been connected to an elevated risk (with a relative risk of 1.55) and grim outlook of liver cancer [6, 14]. This association may be linked to chronic inflammation, cellular apoptosis, and collagen enzyme dysregulation [6, 15–18].

Influenza is a highly contagious seasonal epidemic infection that causes an abrupt onset of high fever, myalgia, and respiratory symptoms [19]. This seasonal infection is responsible for the death of more than 290,000 individuals each year worldwide [19, 20]. It increases hospitalization, morbidity, and mortality rates and places an additional economic burden on patients, especially for senior citizens and individuals with chronic medical conditions [19, 20]. Past statistics also disclosed that virus infection is positive associated with cancer development [21]. Therefore, multiple studies have attempted to clarify the relationship between influenza and cancer and seek strategies to prevent infection.

According to previous studies, influenza vaccination reduces infection, hospitalization, and illness severity [22, 23]. Furthermore, vaccination could reduce the inflammation status and oxidative stress from virus infection [24]. These pathogeneses may also exist in the mechanism of the liver cancer formation [25]. While the exact mechanisms remain unknown, this study focuses on impact of influenza vaccine on the occurrence of liver cancers among a Taiwanese population with hypertension by applying the data from Taiwan’s National Health Insurance Research Database (NHIRD).

## Method

Established in 1995, the National Health Insurance program provides comprehensive healthcare to over 98% of the Taiwanese citizens [26]. This study was conducted using data obtained from the NHIRD (2001–2012). To protect personal privacy, all information obtained from the NHIRD was processed through delinking and deidentifying procedures. During data collection, an agreement was signed to ensure the privacy of the patients’ and care providers’ information. The approval for this study was obtained from the Joint Institutional Review Board of Taipei Medical University (approval no. N201804043).

### Patient selection and definition of the primary endpoint

**Fig. 1:**
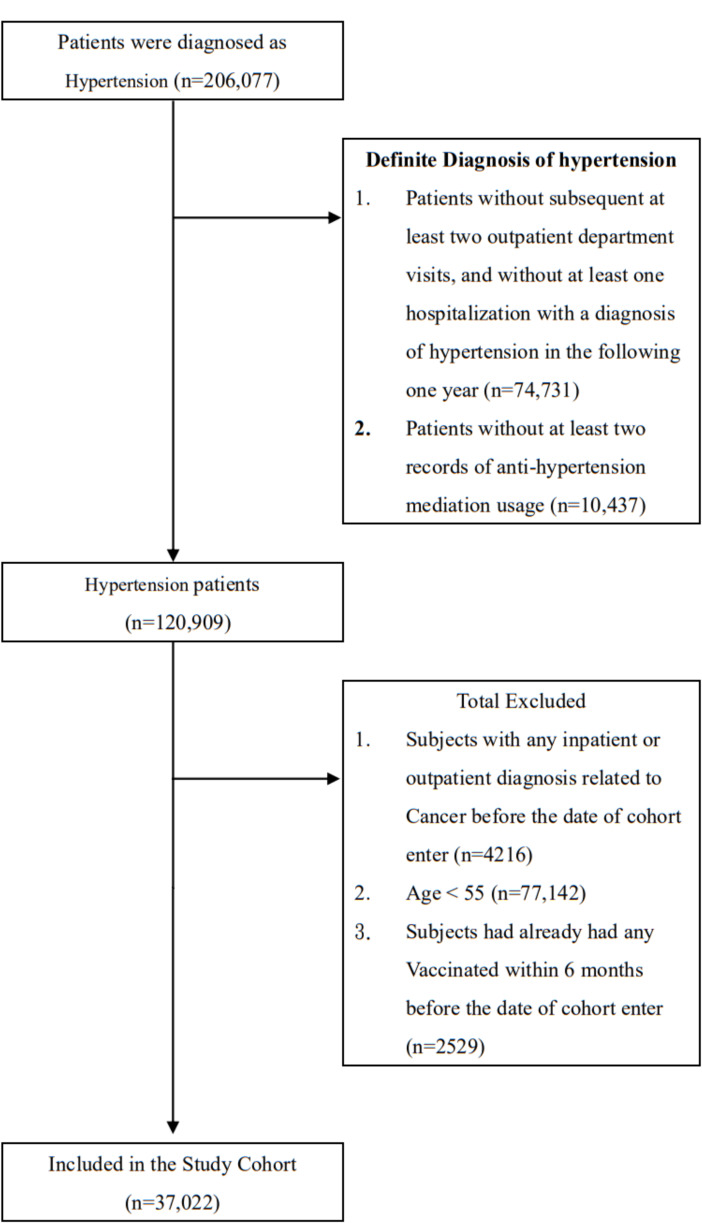
Data selection process

The study cohort included all patients who received a diagnosis of hypertension in accordance with the International Classification of Diseases, Ninth Revision, Clinical Modification (ICD-9-CM, codes 401.X and 402.X) over a period of 12 years from January 1, 2001, to December 31, 2012. Patients without at least two subsequent outpatient department visits and one instance of hospitalization for hypertension in the following year or without at least two records of antihypertension medication use were excluded due to the uncertainty of hypertension diagnosis (Fig. 1). To exclude patients who had cancer before the selection procedure, this study implemented a 1-year (2000) washout period. This study excluded patients under 55 years. As stipulated by the Taiwanese government, vaccination is free for individuals older than 65 years or high-risk individuals aged 50–65 years, specifically individuals who have cardiovascular disease, renal disease, compromised immunity, a body mass index of 30 or greater, or type 2 diabetes mellitus [27]. Besides, in order to eliminate the interference effects caused by recent vaccinations, patients who received influenza vaccination within half a year before the enrollment date were excluded. In this study, we used ICD-9-CM code V048 and vaccine drug codes to examine the vaccination history of our participants. We also set the primary endpoint as the occurrence of primary liver cancer (ICD-9-CM code 155.X) in patients with hypertension during the follow-up period. All patients were followed up until they received a new diagnosis of liver cancer, withdrew from the National Health Insurance program, were lost to follow-up, or died or until December 31, 2012.

### Potential confounders

Possible confounding factors, such as sociodemographic characteristics (age, sex, urbanization level, and monthly income), comorbidities (Charlson Comorbidity Index [CCI], diabetes mellitus [ICD-9-CM, codes 250.X], dyslipidemia [ICD-9-CM, codes 272.X], heart failure [ICD-9-CM, codes 428], acute myocardial infarction [ICD-9-CM, codes 410], atrial fibrillation [ICD-9-CM, codes 427.31], ischemic heart disease [ICD-9-CM, codes 414.9], angina [ICD-9-CM, codes 413], peripheral vascular disease [ICD-9-CM, codes 443.9], cerebrovascular diseases [ICD-9-CM, codes 430–438], renal failure [ICD-9-CM, codes 585.X], hepatitis B virus [ICD-9-CM, codes 070.2X, 070.3X], hepatitis C virus [ICD-9-CM, codes 070.41, 070.44, 070.51, 070.54], metabolic dysfunction-associated fatty liver disease [MAFLD] [ICD-9-CM, codes 571.8, 571.9], cirrhosis [ICD-9-CM, codes 571]), antihypertension medications (antihypertensive drugs [WHO-ATC, codes C02, C02A, C02B, C02C, C02D, C02K, C02L, and C02N], diuretics [WHO-ATC, codes C03], beta blockers [WHO-ATC, codes C07], calcium channel blockers [WHO-ATC, codes C08], and renin–angiotensin–aldosterone system inhibitors [RAASIs] [WHO-ATC, codes C09]), and other medication use (statins [WHO-ATC, codes C10AA], metformin [WHO-ATC, codes A10BA02], and aspirin [WHO-ATC, codes B01AC06 and N02BA01]), were analyzed for each patient included in the study. The reason we select statins, metformin, and aspirin as covariates is that some previous studies have indicated they may have a protective effect against the occurrence of cancer [27–29]. Besides, we categorized the concurrent medications based on the number of the prescription days (< 28 days, 28–365 days, or > 365 days) during the observation period.

### Statistical analysis

**Fig. 2:**
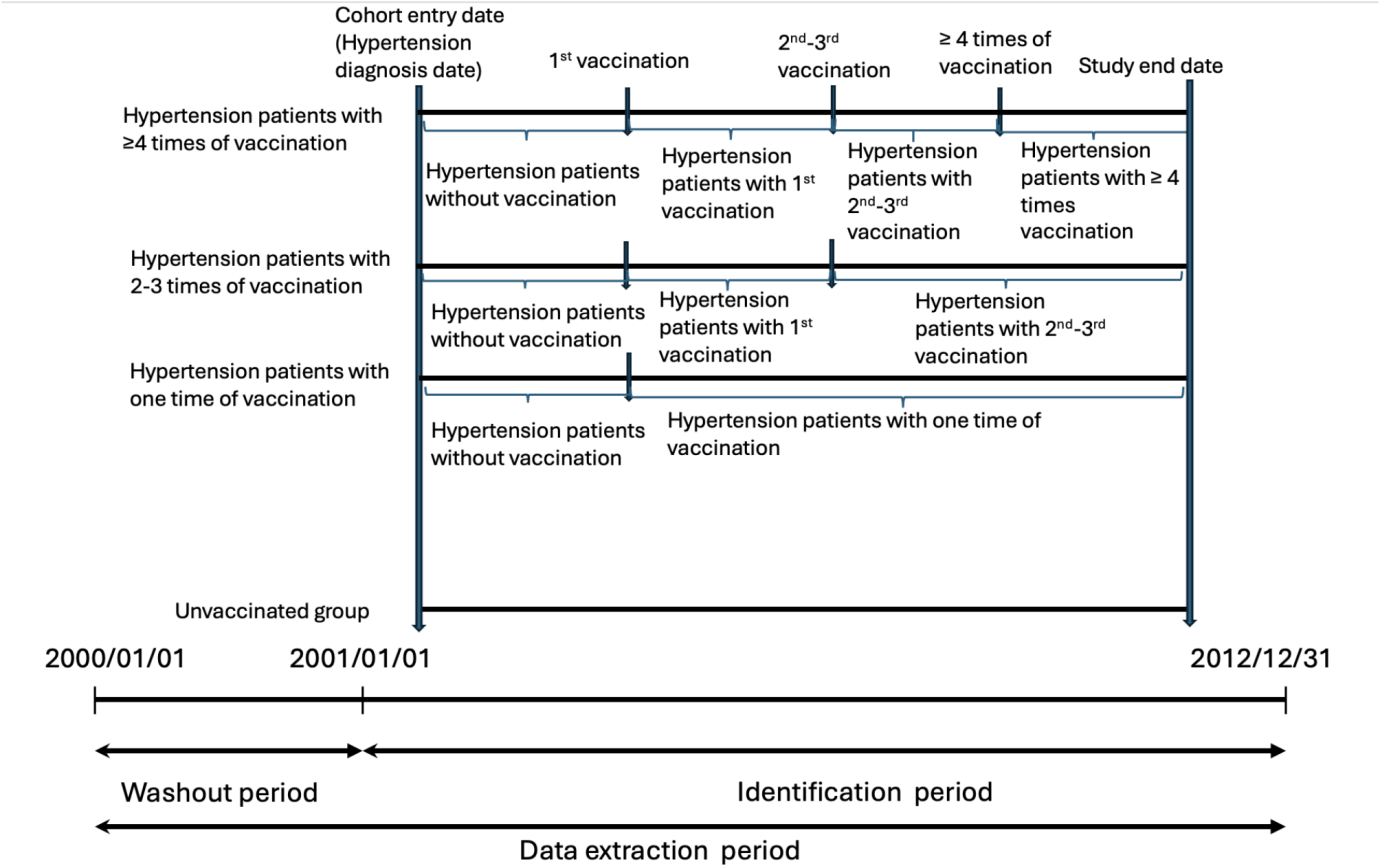
Time-varying analysis in unvaccinated patients and patients who received 1, 2–3 and more than 4 times of vaccination

To minimize selection bias and evaluate the effects of vaccination, we first estimated the baseline propensity scores (PS) using a logistic regression model to predict the probability of receiving influenza vaccine in each patient. In further time-varying logistic regression model analysis, PS adjusting was used to account for covariates between the vaccinated and unvaccinated groups [30, 31]. Categorical and continuous variables were analyzed using the chi-square test and *t* test, respectively. Time-varying COX model was used to evaluate the hazard ratios (HRs) and 95% confidence intervals (CIs) of liver cancer between vaccinated and unvaccinated patients with hypertension. Subsequently, we categorized the hypertension patients into four groups according to their vaccination status (unvaccinated patients, patients who received one vaccine dose, patients who received two or three vaccine doses, and patients who received four or more vaccine doses) to calculate the dose–response effect. The effect of changing vaccination status in each patient was considered. Therefore, a time-varying COX model was used to evaluate the hazard ratios (HRs) and 95% confidence intervals (CIs) of liver cancer between vaccinated and unvaccinated patients with hypertension. Among patients who received vaccines two to three times, the time period before the second vaccination and after the first vaccine would be classified into patients with first-time vaccination. Similarly, among patients who received more than four times of vaccination, the time period would be classified into patients with one time of vaccination and patients with two to three times of vaccination before the second time of vaccination and before the fourth time of vaccination (Fig. 2). Data were then stratified by potential confounding factors, namely age, sex, comorbidities, and medication use. Additionally, we divided the patients into two groups based on the follow-up period: over 10 years and under 10 years. Ultimately, sensitivity analysis was conducted to explore the distinctions and similarities between influenza vaccination and the occurrence of liver cancer in patients with hypertension. All statistical analyses were conducted using IBM SPSS Statistics version 22.0 (IBM, Armonk, NY, USA) and SAS version 9.4 (SAS Institute, Cary, NC, USA). A *p* value less than 0.05 indicated statistical significance.

## Results

### Baseline characteristics

From 2001 to 2012, there were a total of 206,077 patients diagnosed with hypertension. Some patients were excluded due to the uncertainty of the hypertension diagnosis (*n* = 85,168), diagnosis of neoplasm (ICD-9-CM, codes 140–239) before the selection procedure (*n* = 4,216), being under 55 years (*n* = 77,142) and receiving influenza vaccination within half a year before the enrollment date (*n* = 2,529). Finally, there were 37,022 patients included in our study. Among the 37,022 patients with hypertension enrolled in this cohort, 15,697 were vaccinated (42.4%) and 21,325 were unvaccinated (57.6%). The Statistics Power of the study was > 0.999. The two groups significantly differed in terms of age and sex distribution, medication history, and level of urbanization (Table 1). Dyslipidemia, heart failure, acute myocardial infarction, ischemic heart disease, angina, peripheral vascular disease, cerebrovascular diseases, renal failure, hepatitis C virus and cirrhosis was more common among vaccinated than unvaccinated patients (*p* < .001). Comorbidity-associated medications, such as statin, metformin, and aspirin, were used at a lower rate and duration in the unvaccinated than vaccinated group (Table 1).

**Table 1:** Attributes of the Enrolled Population

	All patients (*n* = 37022)		Unvaccination (*n* = 21325)		Vaccination (*n* = 15697)		*P* ^a^
	*n*	%	*n*	%	*n*	%	
*Age*,* years (Mean ± SD)*	66.37 ± 8.08		64.04 ± 8.05		69.52 ± 6.99		< 0.001
55–64	18,511	50.00	13,907	65.21	4604	29.33	< 0.001
65–74	12,618	34.08	4884	22.90	7734	49.27	
≥ 75	5893	15.92	2534	11.88	3359	21.40	
*Sex*							
Female	18,373	49.63	10,378	48.67	7995	50.93	< 0.001
*Charlson Comorbidity Index*							
0	16,843	45.59	9951	46.66	6892	43.91	< 0.001
1	9884	26.70	5622	26.36	4262	27.15	
2	5695	15.38	3213	15.07	2482	15.81	
≥ 3	4600	12.43	2539	11.91	2061	13.13	
*Comorbidities*							
Diabetes	7904	21.35	4542	21.30	3362	21.42	0.782
Hyperlipidemia	28,895	78.05	16,487	77.31	12,408	79.05	< 0.001
HF	6298	17.01	4088	19.17	2210	14.08	< 0.001
AMI	708	1.91	454	2.13	254	1.62	< 0.001
AF	5545	14.98	3280	15.38	2265	14.43	0.011
Ischemic heart disease	14,831	40.06	9070	42.53	5761	36.70	< 0.001
Angina	5011	13.54	3054	14.32	1957	12.47	< 0.001
Peripheral vascular disease	4343	11.73	2725	12.78	1618	10.31	< 0.001
Cerebrovascular diseases	12,180	32.90	5933	27.82	6247	39.80	< 0.001
Renal failure	5284	14.27	3418	16.03	1866	11.89	< 0.001
Hepatitis B virus	690	1.86	412	1.93	278	1.77	0.258
Hepatitis C virus	1449	3.91	749	3.51	700	4.46	< 0.001
MAFLD	7361	19.88	4154	19.48	3207	20.43	0.023
Cirrhosis	1571	4.24	847	3.97	724	4.61	0.003
*Anti-hypertension medications*							
Beta blocking agents	19,043	51.44	10,217	47.91	8826	56.23	< 0.001
Calcium channel blockers	25,891	69.93	14,105	66.14	11,786	75.08	< 0.001
Diuretics	18,450	49.84	9318	43.70	9132	58.18	< 0.001
RAASI	22,178	59.90	11,896	55.78	10,282	65.50	< 0.001
Other antihypertensive drugs	7154	19.32	3291	15.43	3863	24.61	< 0.001
*Co-drugs*							
Statin drugs							
< 28 days	27,284	73.70	16,085	75.43	11,199	71.34	< 0.001
28–365 days	6124	16.54	3472	16.28	2652	16.89	
>365 days	3614	9.76	1768	8.29	1846	11.76	
Metformin drug							
< 28 days	29,674	80.15	17,300	81.13	12,374	78.83	< 0.001
28–365 days	2797	7.55	1724	8.08	1073	6.84	
>365 days	4551	12.29	2301	10.79	2250	14.33	
Aspirin drug							
< 28 days	21,745	58.74	13,765	64.55	7980	50.84	< 0.001
28–365 days	8458	22.85	4511	21.15	3947	25.14	
>365 days	6819	18.42	3049	14.30	3770	24.02	
*Level of Urbanization*							
Urban	25,030	67.61	15,421	72.31	9609	61.22	< 0.001
Suburban	7992	21.59	4146	19.44	3846	24.50	
Rural	4000	10.80	1758	8.24	2242	14.28	
*Monthly income (NT$)*							
0	4327	11.69	2150	10.08	2177	13.87	< 0.001
1-19200	11,477	31.00	6078	28.50	5399	34.40	
19,200–25,000	11,832	31.96	6066	28.45	5766	36.73	
≥25,001	9386	25.35	7031	32.97	2355	15.00	

^a^ Comparison between Unvaccination and Vaccination

HF, heart failure, AMI, acute myocardial infarction, AF, atrial fibrillation, MAFLD, metabolic dysfunction-associated fatty liver disease, RAASI, renin-angiotensin-aldosterone system inhibitor

### HRs of liver cancer in the vaccinated and unvaccinated groups

Table 2 demonstrates the incidences of liver cancer in patients with hypertension as distinguished by vaccination status. After potential confounders were adjusted for, the results indicated that the incidence of liver cancer was notably reduced in the vaccinated group compared to the unvaccinated group (Incidence rate of unvaccinated = 345.7, Incidence rate of vaccinated = 179.1; HR = 0.56, 95% CI = 0.46–0.64; *p* < .001). The protective effect was consistent across different age and sex groups, the HR was 0.53 in women (95% CI = 0.39–0.68; *p* < .001) and 0.58 in men (95% CI = 0.44–0.74; *p* < .001).

**Table 2:** Risk of Liver Cancer among Unvaccinated and Vaccinated in Study Cohort

All patients (*N* = 37022)	Unvaccinated (Total follow-up 96626.9 person-years)				Vaccinated (Total follow-up 94366.2 person-years)				Adjusted HR† (95% C.I.)
	No. of Patients With Cancer	Incidence Rate (per 10^5^ person-years) (95% C.I.)			No. of Patients With Cancer	Incidence Rate (per 10^5^ person-years) (95% C.I.)			
**Whole cohort**									
liver cancer	334	345.7	(308.6,	382.7)	169	179.1	(152.1,	206.1)	0.56(0.46, 0.64)***
**Age**,** 55-64**^a^									
liver cancer	196	314.8	(270.8,	358.9)	38	125.0	(85.3,	164.8)	0.50(0.34, 0.69)***
**Age**,** 65-74**^b^									
liver cancer	94	401.2	(320.1,	482.3)	90	196.0	(155.5,	236.5)	0.55(0.40, 0.72)***
**Age**,** ≥ 75**^c^									
liver cancer	44	402.1	(283.3,	521.0)	41	227.0	(157.5,	296.4)	0.57(0.39, 0.82)**
**Female** ^d^									
liver cancer	136	279.1	(232.2,	326.0)	70	143.3	(109.7,	176.8)	0.53(0.39, 0.68)***
**Male** ^e^									
liver cancer	198	413.4	(355.8,	470.9)	99	217.6	(174.7,	260.4)	0.58(0.44, 0.74)***

^a^Total follow-up 62254.4 person-year for unvaccinated and 30393.7 for Vaccinated

^b^Total follow-up 23430.8 person-year for unvaccinated and 45908.6 for Vaccinated

^c^Total follow-up 10941.7 person-year for unvaccinated and 18063.9 for Vaccinated

^d^Total follow-up 48727.6 person-year for unvaccinated and 48864.1 for Vaccinated

^e^Total follow-up 47899.3 person-year for unvaccinated and 45502.1 for Vaccinated

C.I.: confidence interval *: *P* < .05 **: *P* < .01 ***: *P* < .001

HR: hazard ratio

**†**Main model is adjusted for Age, Sex, CCI Index, Diabetes, Hyperlipidemia, HF, AMI, AF, Ischemic heart disease, Angina, Peripheral vascular disease, Cerebrovascular diseases, Renal failure, Hepatitis B virus, Hepatitis C virus, Cirrhosis, Beta blocking agents, Calcium channel blockers, Diuretics, RAASI, Other antihypertensive drugs, Statin, Metformin, Aspirin, level of urbanization, monthly income in propensity score

### Sensitivity analysis of the relationship between the number of vaccine doses and the risk of liver cancer

As shown in Table 3, sensitivity analysis was conducted with covariate adjustments, and the vaccinated group was stratified by the total number of vaccine doses. The result revealed not only the decreased HR of liver cancer in different groups after vaccination, but also the dose-dependent protective effects. When more than two vaccine doses were administered, the risk of liver cancer significantly decreased (Incidence Rate = 214.8, adjusted HR = 0.60, 95% CI = 0.51–0.78; Incidence Rate = 173.6, adjusted HR = 0.48, 95% CI = 0.38–0.65; and Incidence Rate = 128.1, adjusted HR = 0.39, 95% CI = 0.30–0.51 for one dose, two or three doses, and four or more doses, respectively).This protective effect was also observed in individuals with comorbidities, including those with a high CCI index, diabetes, and dyslipidemia. Table 4 demonstrated the risk of liver cancer among unvaccinated and vaccinated in study cohort stratified into 10-year follow-up periods.

**Table 3:** Sensitivity analysis of adjusted HRs of vaccination in risk reduction of Liver Cancer

	Unvaccinated	Vaccinated			*P* for Trend
		1	2–3	≥ 4	
	Adjusted HR (95%C.I.)	Adjusted HR (95%C.I.)	Adjusted HR (95%C.I.)	Adjusted HR (95%C.I.)	
**Follow-up duration** **(person-years)**	96626.9	35842.9	37444.8	21078.5	
**Number of Events**	334	77	65	27	
**Incidence Rate** **(per 10** ^**5**^ **person-years)**	345.7	214.8	173.6	128.1	
**Main model†**	1.00	0.60(0.51, 0.78)**	0.48(0.38, 0.65)***	0.39(0.30, 0.51)***	< 0.001
**Main model† + MAFLD**	1.00	0.59(0.50, 0.77)**	0.47(0.37, 0.64)***	0.38(0.30, 0.50)***	< 0.001
Age, years					
55–64	1.00	0.43(0.29, 0.75)*	0.42(0.28, 0.70)**	0.49(0.29, 0.98)*	0.086
65–74	1.00	0.81(0.55, 1.13)	0.45(0.34, 0.67)***	0.31(0.24, 0.45)***	< 0.001
≥ 75	1.00	0.52(0.29, 0.99)*	0.55(0.33, 0.94)*	0.41(0.22, 0.71)**	< 0.001
Sex					
Female	1.00	0.60(0.40, 0.88)*	0.45(0.32, 0.64)***	0.35(0.26, 0.52)***	< 0.001
Male	1.00	0.61(0.41, 0.87)*	0.52(0.39, 0.73)***	0.44(0.33, 0.61)***	< 0.001
CCI Index^+^					
0	1.00	0.42(0.26, 0.86)*	0.59(0.40, 0.90)*	0.37(0.27, 0.60)***	0.034
1	1.00	0.75(0.45, 1.28)	0.32(0.19, 0.59)***	0.41(0.30, 0.69)***	< 0.001
2	1.00	0.67(0.42, 1.24)	0.52(0.31, 0.91)*	0.48(0.33, 0.81)*	< 0.001
≥ 3	1.00	0.68(0.43, 1.26)	0.44(0.24, 0.79)**	0.27(0.20, 0.49)***	< 0.001
Diabetes					
No	1.00	0.58(0.42, 0.84)*	0.43(0.32, 0.69)***	0.40(0.30, 0.54)***	< 0.001
Yes	1.00	0.64(0.35, 1.13)	0.60(0.45, 1.01)	0.37(0.26, 0.64)***	< 0.001
Hyperlipidemia					
No	1.00	0.55(0.41, 0.80)*	0.50(0.38, 0.66)***	0.40(0.30, 0.53)***	< 0.001
Yes	1.00	0.86(0.47, 1.51)	0.42(0.23, 0.85)*	0.35(0.25, 0.60)***	< 0.001
Hepatitis B virus					
No	1.00	0.63(0.49, 0.87)**	0.47(0.37, 0.64)***	0.40(0.32, 0.53)***	< 0.001
Yes	1.00	0.54(0.21, 0.96)*			-
Hepatitis C virus					
No	1.00	0.63(0.51, 0.89)**	0.45(0.36, 0.61)***	0.40(0.30, 0.51)***	< 0.001
Yes	1.00	0.50(0.23, 1.96)	0.47(0.18, 2.14)		0.621
Cirrhosis					
No	1.00	0.65(0.52, 0.90)*	0.46(0.37, 0.62)***	0.39(0.30, 0.51)***	< 0.001
Yes	1.00	0.46(0.20, 1.77)	0.44(0.25, 2.06)		0.596
Other antihypertensive drugs					
No (< 28 days)	1.00	0.60(0.45, 0.80)*	0.42(0.32, 0.58)***	0.38(0.29, 0.52)***	< 0.001
Yes (≥ 28 days)	1.00	0.68(0.37, 1.53)	0.87(0.50, 1.61)	0.41(0.30, 0.55)**	0.095
Diuretics					
No (< 28 days)	1.00	0.61(0.46, 0.92)*	0.40(0.30, 0.58)***	0.29(0.20, 0.45)***	< 0.001
Yes (≥ 28 days)	1.00	0.60(0.44, 0.96)*	0.59(0.42, 0.88)*	0.46(0.36, 0.67)***	< 0.001
Beta blocking agents					
No (< 28 days)	1.00	0.62(0.42, 0.96)*	0.48(0.32, 0.71)***	0.32(0.23, 0.46)***	< 0.001
Yes (≥ 28 days)	1.00	0.59(0.41, 0.94)*	0.48(0.32, 0.70)***	0.43(0.32, 0.65)***	< 0.001
Calcium channel blockers					
No (< 28 days)	1.00	0.50(0.31, 0.85)*	0.40(0.24, 0.64)***	0.36(0.28, 0.53)***	< 0.001
Yes (≥ 28 days)	1.00	0.68(0.50, 1.02)	0.55(0.39, 0.76)***	0.40(0.31, 0.56)***	< 0.001
RAASI					
No (< 28 days)	1.00	0.59(0.40, 0.90)*	0.50(0.35, 0.74)***	0.39(0.29, 0.52)***	< 0.001
Yes (≥ 28 days)	1.00	0.61(0.41, 0.92)*	0.47(0.32, 0.70)***	0.40(0.30, 0.54)***	< 0.001
Statin drugs					
<28 days	1.00	0.66(0.49, 0.91)*	0.45(0.34, 0.68)***	0.39(0.28, 0.54)***	< 0.001
28–365 days	1.00	0.35(0.19, 1.07)	0.34(0.16, 0.83)*	0.30(0.18, 0.66)**	< 0.001
>365 days	1.00	0.62(0.24, 4.23)	1.14(0.41, 3.97)	0.41(0.08, 1.90)	0.415
Metformin drug					
< 28 days	1.00	0.65(0.49, 0.90)*	0.43(0.34, 0.59)***	0.39(0.30, 0.54)***	< 0.001
28–365 days	1.00	0.45(0.24, 1.26)	0.44(0.25, 0.63)**		0.041
>365 days	1.00	0.66(0.45, 1.81)	0.91(0.60, 1.83)	0.54(0.42, 0.76)*	0.201
Aspirin drug					
< 28 days	1.00	0.67(0.50, 0.94)*	0.43(0.33, 0.64)***	0.33(0.23, 0.48)***	< 0.001
28–365 days	1.00	0.49(0.23, 0.99)*	0.40(0.25, 0.72)**	0.39(0.26, 0.57)***	< 0.001
>365 days	1.00	0.48(0.15, 1.36)	0.82(0.49, 1.67)	0.57(0.42, 1.23)	0.232

C.I.: confidence interval *: *P* < .05 **: *P* < .01 ***: *P* < .001

HR: hazard ratio

**†**Main model is adjusted for Age, Sex, CCI Index, Diabetes, Hyperlipidemia, HF, AMI, AF, Ischemic heart disease, Angina, Peripheral vascular disease, Cerebrovascular diseases, Renal failure, Hepatitis B virus, Hepatitis C virus, Cirrhosis, Beta blocking agents, Calcium channel blockers, Diuretics, RAASI, Other antihypertensive drugs, Statin, Metformin, Aspirin, level of urbanization, monthly income in propensity score

MAFLD, Metabolic Dysfunction-Associated Fatty Liver Disease

**Table 4:** Risk of Liver Cancer among Unvaccinated and Vaccinated in Study Cohort stratified into 10-year follow-up periods

	All patients		< 10 years		> 10 years	
	follow-up	Cancer	follow-up	Cancer	follow-up	Cancer
Unvaccinated	96626.9	334	66462.6	320	30164.3	14
Vaccinated	94366.2	169	72288.8	158	22077.4	11
1	35842.9	77	23476.0	76	12366.9	1
2–3	37444.8	65	27734.2	55	9710.6	10
**≥** 4	21078.5	27	21078.5	27	-	-
Adjusted HR1	0.56(0.46, 0.64)***		0.45(0.35, 0.73)***		1.02(0.68, 1.81)	
Adjusted HR2	0.60(0.51, 0.78)**		0.67(0.53, 0.94)*		0.17(0.04, 1.36)	
Adjusted HR3	0.48(0.38, 0.65)***		0.41(0.29, 0.57)***		2.01(0.90, 5.26)	
Adjusted HR4	0.39(0.30, 0.51)***		0.26(0.17, 0.51)***		-	

C.I.: confidence interval *: *P* < .05 **: *P* < .01 ***: *P* < .001

HR: hazard ratio

**†**Main model is adjusted for Age, Sex, CCI Index, Diabetes, Hyperlipidemia, HF, AMI, AF, Ischemic heart disease, Angina, Peripheral vascular disease, Cerebrovascular diseases, Renal failure, Hepatitis B virus, Hepatitis C virus, Cirrhosis, Beta blocking agents, Calcium channel blockers, Diuretics, RAASI, Other antihypertensive drugs, Statin, Metformin, Aspirin, level of urbanization, monthly income in propensity score

## Discussion

### Major findings

The key discoveries of this cohort study based on the population are as follows. First, patients with hypertension who received influenza vaccine had a low incidence of liver cancer. Second, the larger the number of vaccine doses administered was, the greater the reduction in the risk of liver cancer was.

### Mechanism of liver cancer development in patients with hypertension

According to previous studies, patients with hypertension have an increased risk of liver cancer [6]. Several candidate mechanisms underlying the association between hypertension and liver cancer have been proposed. One of these mechanisms is the renin–angiotensin–aldosterone system (RAAS), which is a crucial vasoactive factor that regulates blood pressure. This system exists in both the circulatory system and local tissues, including the heart, liver, and kidneys [18, 32]. RAAS dysregulation results in not only hypertension but also proinflammatory and profibrotic sequelae, with the production of an intermediate product. These sequelae increase the risk of liver fibrosis and hepatocarcinogenesis [32]. Furthermore, patients with hypertension exhibit increased vascular endothelial growth factor (VEGF) expression. VEGF contributes to cancer progression by affecting blood vessel permeability, proliferation, and endothelial cell growth [6, 33]. Another mechanism linking hypertension to liver cancer is metabolic syndrome, which, similar to hypertension, stimulates the production of reactive oxygen species (ROS) and results in inflammation. These sequelae may influence the regulation of the extracellular matrix (ECM) and tissue repair, induce a malignant phenotype, and trigger tumor development [6, 34, 35]. Matrix metalloproteinases (MMPs), a group of enzymes that degrade collagen in the ECM, also serve as a link between hypertension and cancer. Recent studies have indicated a correlation between MMPs and hypertension through insulin resistance and mechanical stress [34, 36]. The stiffening and degradation of the ECM and the regulation of inflammation by MMPs play a role in the development of cancer [37].

### Relationship between the risk of liver cancer and influenza vaccination

The primary purpose of vaccination is to provide protection against infection or severe illness. Additionally, it is be pointed that human papillomavirus vaccine and hepatitis B vaccine also prevent people from cancer. Influenza vaccination has also been reported to be associated with a lower risk of lung cancer [38]. In patients with influenza, the number of inflammatory cytokines increases in their circulatory system, resulting in cellular necrosis and oncogenic signaling cascades [18, 39]. This process also induces inflammation and ROS production, which not only cause cellular damage but also affect the ECM, as is the case in hypertension [38, 40]. Consequently, cell proliferation, polarity, differentiation, migration, and adhesion are all affected in the ECM [6]. Inappropriate remodeling of the ECM may exacerbate the risk of cancer [41]. In addition, MMPs play a role in immune responses to infection. However, MMP overactivation following infection may increase the host’s vulnerability to pathogen attack, tissue damage, and proto-oncogene activation [6, 42]. Hence, vaccination against influenza may protect patients from infection and liver cancer.

### Effect of influenza vaccination in different subgroups

Overall, our findings suggested that influenza vaccination has a stronger protective effect in women than in men. According to a previous study, women have a lower risk of liver cancer than men presumably because estrogen reduces the concentration of inflammatory cytokines [43]. Both estrogen and influenza vaccine may have a synergic effect that increases the level of protection. Patients who take blood pressure medication over a short period have a low risk of liver cancer because their hypertension may be less severe than patients who do not take such medication. Thus, vaccination can effectively control inflammation and the production of ROS and MMPs. However, the dose-dependent effect of influenza vaccine seems to be more pronounced in individuals aged above 65, presumably because the immune system tends to weaken with age, making it less responsive to vaccines [44, 45]. Consequently, additional vaccine doses may be required to stimulate memory cell function. As a result, implementing a consistent and well-planned vaccination policy is imperative for elderly individuals with hypertension.

### Limitations

This study has some limitations. First, we identified patients with hypertension on the basis of ICD-9-CM codes, which may have resulted in some misclassification. Thus, we incorporated drug, outpatient, and hospitalization data for greater accuracy. Second, the NHIRD does not report on personal behavioral data (e.g., cigarette and alcohol use), blood pressure data, or biochemical data. We were also unable to differentiate between different types of liver cancer, such as hepatocellular carcinoma and cholangiocarcinoma, to evaluate the effect of vaccination on each category. However, we discovered that vaccination against influenza was associated with a reduced risk of liver cancer. We also performed PS adjusting to minimize bias due to potential confounders. Third, because of vaccination policies in Taiwan, we only enrolled patients aged above 55. Therefore, our experimental results may not be generalizable to all age groups. Fourth, this was not a prospective randomized blinded study and thus could not provide causal evidence. Fifth, the chosen cohort for this study spans from 2001 to 2012. It is necessary to analyze more recent data in future studies to confirm the findings of the current research. In addition, there were limited patients with a follow-up period more than 10 years, future study is warranted for validating the long-term effect of vaccine.

## Conclusion

In hypertensive patients, getting vaccinated against influenza is linked to a lowered risk of liver cancer. However, additional research is required to investigate the mechanism underlying this association.

## Data Availability

Data are not accessible as a result of regulations imposed by the Taiwan National Health Insurance Research Database (NHIRD).

## Abbreviations

NHIRD: National Health Insurance Research Database
CCI: Charlson Comorbidity Index
HF: Heart failure
AMI: Acute myocardial infarction
AF: Atrial fibrillation
RAASIs: Renin–angiotensin–aldosterone system inhibitors
PS: Propensity score
HRs: Hazard ratios
Cis: Confidence intervals
RAAS: Renin–angiotensin–aldosterone system
VEGF: Vascular endothelial growth factor
ROS: Reactive oxygen species
ECM: Extracellular matrix
MMPs: Matrix metalloproteinases
